# Multispectral Imaging for Determination of Astaxanthin Concentration in Salmonids

**DOI:** 10.1371/journal.pone.0019032

**Published:** 2011-05-10

**Authors:** Bjørn S. Dissing, Michael E. Nielsen, Bjarne K. Ersbøll, Stina Frosch

**Affiliations:** 1 Department of Informatics and Mathematical Modeling, Technical University of Denmark, Kgs. Lyngby, Denmark; 2 Division of Industrial Food Technology, National Food Institute, Technical University of Denmark, Kgs. Lyngby, Denmark; Institute of Marine Research, Norway

## Abstract

Multispectral imaging has been evaluated for characterization of the concentration of a specific cartenoid pigment; astaxanthin. 59 fillets of rainbow trout, *Oncorhynchus mykiss*, were filleted and imaged using a rapid multispectral imaging device for quantitative analysis. The multispectral imaging device captures reflection properties in 19 distinct wavelength bands, prior to determination of the true concentration of astaxanthin. The samples ranged from 0.20 to 4.34 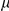
g per g fish. A PLSR model was calibrated to predict astaxanthin concentration from novel images, and showed good results with a RMSEP of 0.27. For comparison a similar model were built for normal color images, which yielded a RMSEP of 0.45. The acquisition speed of the multispectral imaging system and the accuracy of the PLSR model obtained suggest this method as a promising technique for rapid in-line estimation of astaxanthin concentration in rainbow trout fillets.

## Introduction

Color is a highly important quality parameter in relation to the commercial production of salmonid fishes. The consumers associate increased intensity of red in salmonid fishes with superior quality, being fresher and having a better flavor [1], [2]. As the change in surface color is the first quality parameter evaluated by the consumer, it is of great economic importance that the color of the salmonid fishes meets consumer preferences. The color of salmonid fishes is caused by deposition of cartenoid pigments in the muscular tissue. Besides being essential for reproduction, proper growth and survival of the fish, carotenoids, primarily astaxanthin and castaxanthin, are also important for the red color in salmonids. As fish cannot synthesize carotenoids de novo their intake rely on the content of cartenoids in the feed. Wild salmonids obtain the cartonids from intake of e.g. crusteceans, krill and other sources rich in carotenids whereas carotenoids primarily astaxanthin is added to the feed of farmed salmonids. The primary use of astaxanthin within aquaculture is as a feed additive to ensure that farmed salmon and trout achieve a coloration that comply with the consumers preferences.

Astaxanthin is the single most expensive constituent in salmonid fish feed. Even though astaxanthin constitutes less than 20% of the total fish feed costs, control and optimization of the concentration of astaxanthin from feed to fish is of paramount importance for a cost effective salmonid fish production. Traditionally, astaxanthin content in fish is determined by spectrophotometric analysis or high-performance liquid chromatography (HPLC) analysis. In both methods astaxanthin is extracted from the minced sample into a suitable solvent such as acetone or hexane before further analysis. U.S. Food and Drug Administration (21 CFR 73.185) and Canadian Food Inspection Agency (Registration no. 990535) have accepted the method based on HPLC analysis for determining astaxanthin content of a product. Both methods have several drawbacks. First, the method based on spectrophotometric analysis overestimate the astaxanthin as other compounds such as lutein, canthaxanthin and astacene are falsely included. This means they absorb light at the same wavelength as astaxanthin and thereby increase the signal. Second, both methods are time consuming, labor demanding and sample destructive.

For quality assessment of salmonid color there are two widely accepted color standards in the salmonid industry, which are used by quality inspectors in their visual assessment of fillets, the SalmoFan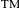 card and the SalmoCard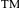 (Hoffmann-La Roche Basel, Switzerland). Both methods enable an inspector to score the color of a salmonid fillet into one of 15 red color categories ranging from 20 (pale red) to 34 (dark red). This method has the advantage of being a very straight forward, intuitive and cheap. It is easily applicable and does require intensive expert training. In spite of these advantages there may be reasons to inspect the color quality of fish fillets using other methods. A human operator is required in order to use the SalmoFan/SalmoCard, which means such a color-evaluation will be subject to operator bias and fatigue while also being time-consuming, costly and relatively labor-expensive.

Other instruments previously used for color evaluation are tricolorimeters, spectrophotometers and standard trichromatic charged coupled device cameras. These devices probe the visual spectrum in order to in some sense imitate human visual perception and objectively quantify colors. A Colorimeter (e.g. Minolta Chroma Meter II-CR200, Hunterlab Miniscan) makes use of a stable light source such as Xenon to illuminate a small surface patch of roughly 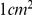, and measures the reflection of the surface in this area. The reflection is then integrated according to the CIE-XYZ [3] tristimulus curves and transformed to the uniform L*, a*, b* color space [3]. The L*, a*, b* color space is a three dimensional color space, where L* represents the lightness of the color (100 being diffuse white), a* the mix of red and green and b* the mix of yellow and blue. Examples of studies where a colorimeter was used in conjunction with studies of fish color include [4]–[6] where the latter established that the intensity of redness (a*) increases with the carotenoid content in the raw flesh of Atlantic salmon, while lightness (L*) decreases and yellowness (b*) remains unaffected. While colorimeters acquire very accurate colors, they do not contain any spatial information, and therefore no information on surface texture and structure/shape. On the other hand chromatic images measure a larger spatial area of reflected photons and thereby provide color as well as spatial information. A review of vision technology and color cameras in the food industry may be found in [7]. The actual color evaluation ability of a trichromatic camera in regard to fish quality inspection was investigated in [8], where comparisons between trichromatic camera images and SalmoFan evaluations were performed on fillets of the Atlantic salmon (Salmo salar). The comparison was based on measurements from five different locations, more or less uniformly spatially distributed across the fillet surface. Here the authors found that there was no significant statistical difference between SalmoFan and camera-based evaluations. Similar experiments were performed in [9], where the authors found a correlation of 0.95 between sensory panel SalmoFan evaluations and computer vision based color evaluations. Current state-of-the art vision systems for quality and process control in the fish processing industries are typically based on traditional trichromatic (Red Green Blue) imaging. In this study we are interested in going one step further, by quantifying the astaxanthin content and thereby indirectly also the color of the fillet. The relative presence of some wavelengths and absence of others is a specific characteristic of many material properties. Consequently the adaption of multispectral imaging technology can reveal relevant information and measurement of more biological quality parameters such as fat, astaxanthin and cartilage content, simultaneously. A multispectral image may also be referred to as a surface chemistry map [10] where a set of neighboring spectra are recorded, revealing information about the surface chemistry to a larger degree than in a trichromatic image. Thus, multispectral imaging is well suited for applications where it is crucial to detect small differences in texture, color and surface chemistry [11]–[15]. It is expected that vision systems based on multispectral imaging will be employed to a much larger extent in the near future [16]–[20]. Aquaculture and the fish processing industries are areas where the added information in a multispectral image can be exploited to improve the general quality and/or reduce the production cost. In this study we investigate the use of multispectral images for estimating natural astaxanthin concentration in rainbow trout fillets. In order to justify the use of multispectral images we calibrate and compare multivariate models for multispectral as well as traditional color images of trout fillets to predict astaxanthin content. Furthermore we illustrate shortly how the calibrated model can be used to predict all spectra in an image in a pixel-wise manner, in order to visualize the predicted spatial astaxanthin distribution within the fish fillet.

## Materials and Methods

### Sample preparation

**Figure 1:**
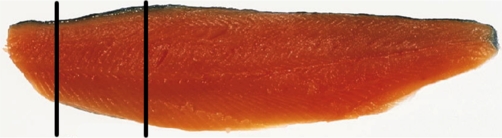
Trout fillet. This image shows how the fillets were cut in order to fit under the camera. The middle piece is used in further analysis.

The Rainbow trout (*Oncorhynchus mykiss*) were from the organic farm at Bisserup Havbrug and harvested in November 2009. The fish were fed with commercial organic trout feed of approximately 1.5% of body weight per day throughout the entire rearing in accordance with commercial practise. According to legislation, the fish feed were coated with natural astaxanthin [21]. The fish were slaughtered at 2 years of age, with an average weight of 1.1 kg. The fish were filleted and trimmed by hand at Bisserup Havbrug the day after slaughtering and transported to the Technical University of Denmark on ice at the same day. The fillets were stored overnight on ice in a 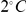 chill room. The fillets were then subsequently cut into three pieces (Figure 1). The middle piece was used as the experimental sample in the further analysis. The samples were placed in plastic petri dishes (90 mm diameter) and stored on ice in styrofoam boxes. Multispectral images of the samples were captured 30 minutes after placement in the styrofoam box. Right after image capture each sample was minced and subsequently frozen at 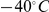. After 14 days of storage at 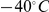 the astaxanthin concentrations were determined using chemical extraction.

### Chemical determination of astaxanthin content

Astaxanthin content of the minced fillets was determined in duplicate from the lipid extracts of the fish meat using an Agilent 1100 series HPLC (Agilent Technologies, Palo Alto, CA), equipped with a UV diode array detector. The fillet sample were minced, and 10 g in duplicates was used for extraction using chloroform and methanol according to the modified protocol of Bligh and Dyer [22]. A fraction of the lipid extract was evaporated under nitrogen and redissolved in 2 mL of n-heptane before injection.Astaxanthin content was determined after injection of an aliquot (50 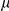
L) of the n-heptane fraction onto a LiChrosorb Si60-5 column (100 mm ×3 mm, 5 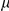
m) equipped with a Cromsep Silica (S2) guard column (10 mm ×2 mm; Chrompack, Middelburg, The Netherlands) and eluted with a flow of 1.2 mL min-1 using n-heptane/acetone (86:14, v/v) and detection at 470 nm. Concentrations of astaxanthin were calculated using authentic standards from Dr. Ehrenstprfer GmbH (Augsburg, Germany).

### Reflection characteristics of astaxanthin

The reflection properties of natural astaxanthin [22] in a solution of fishoil was recorded by a NIRSystems 6500 absorption spectrometer and transformed to reflection values using the standard relation 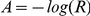, where 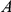 is absorption values and 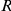 is the reflection values.

### Multispectral Imaging System

**Figure 2:**
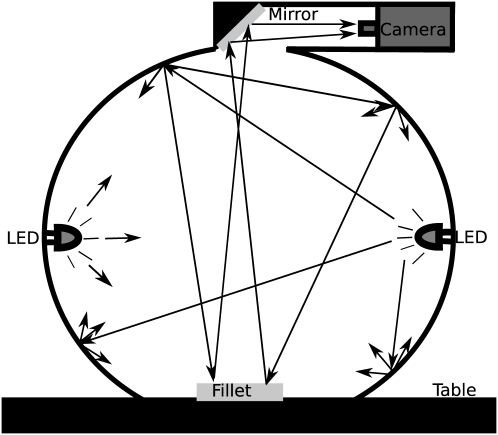
Principal setup of the multispectral system. An integrating sphere coated with a matte white coating ensures optimal lighting conditions. In the rim of the sphere a set of narrow band light emitting diodes ranging from 395 to 970 nm. are mounted. The image acquisition is performed by a monochrome grayscale CCD camera mounted in the top of the sphere. The arrows illustrate how the light is distributed inside the sphere to uniformly illuminate the fillet.

Data acquisition was done using a VideometerLab [10], which obtains multi-spectral images at 19 different wavelengths ranging from 385 to 970 nm, fully shown in Table 1. The acquisition system records surface reflections with a standard monochrome charged coupled device chip, nested in a Point Grey Scorpion camera. Figure 2 shows the principal setup of the system where the object of interest is placed inside an integrating or so called Ulbricht sphere, with a matte white coating. The coating, together with the curvature of the sphere, ensures a uniform reflection of the cast light and thereby a uniform light in the entire sphere. At the rim of the sphere Light Emitting Diodes (LED) are positioned side by side in a pattern which distributes the LEDs belonging to each wavelength uniformly around the entire rim. The system is first calibrated radiometrically using both a diffuse white and dark target followed by a light setup based on the type of object to be recorded. The system is geometrically calibrated with a geometric target to ensure pixel correspondence for all spectral bands [23]. The homogeneous diffuse light, together with the calibration steps, ensures an optimal dynamic range and minimizes shadows and shading effects as well as specular reflection and gloss-related effects. The system has been developed to guarantee the reproducibility of collected images which means it can be used in comparative studies of time series or across a large variety of different samples [24]–[27].

**Table 1:** Spectral bands of VidemeterLab.

01) 395 nm.	09) 630 nm.	17) 940 nm.
02) 435 nm.	10) 645 nm.	18) 950 nm.
03) 450 nm.	11) 660 nm.	19) 970 nm.
04) 470 nm.	12) 700 nm.	
05) 505 nm.	13) 850 nm.	
06) 525 nm.	14) 870 nm.	
07) 570 nm.	15) 890 nm.	
08) 590 nm.	16) 910 nm.	

Narrowbanded lightsources of VideometerLab. The wavelength values shown are the peak values of all light emitting diodes mounted in the sphere. The diodes cover the visible and the first part of the near infrared spectrum.

### Color Images

The advantage of going from color to multispectral images is illustrated by comparing models calibrated using either of the two types of images. To be able to compare results from the two models we have transformed the multispectral images to RGB images. In this paper we used a spectral reconstruction technique [28] in order to estimate the reflectance spectrum in each pixel with 5 nm spacing. Each spectrum was then integrated over the entire recorded spectral range in 3 different intervals, according to a CIE 1931 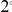 Standard Observer [3]. The resulting color images were then transformed to standard RGB images using a transformation formula described by Wyszecki, G. and Stiles [3].

### Image segmentation and data extraction

Segmenting images into distinct regions is a very important preprocessing step in image analysis before further analyzing the images. Having a specific region representing only the area of the image which should be analyzed is called a region of interest (ROI). Segmentation of images may be done in a large variety of ways, where we in this work made use of statistical orthogonal methods or so called decomposition techniques to highlight desired features for easy extraction. Specifically we have used a Maximum Noise Fraction (MNF) [29] transformation to remove the image background material (petri dish and cardboard under the petri dish). Canonical Discriminant Analysis (CDA) [30] was then used to remove areas assumed to be fat and collagen. The decomposed result with desired features highlighted was then segmented easily using an adaptive thresholding technique known as Otsus adaptive thresholding method [31]. Having segmented the image into a ROI, the image could be transformed to a spectrum based on a mean calculation. Thus each image contributed with a single spectrum for the model calibration.

### Data Analysis

Partial least square regression (PLSR) [32] was used to estimate calibration models between the extracted spectra and reference values (chemically determined) using LOOCV; a total of 59 samples were included in the analysis; 20 for training and 39 for validation. Models were calibrated using leave one out cross validation (LOOCV) [33] using a training set and validated using a testset. The quality of the models was determined based on the coefficient of determination (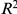), the prediction error expressed as the root mean square error of prediction (RMSEP) and the standard error of the fit. Spectra were centered by substracting the mean calculated from each wavelength followed by a scaling with the standard deviation calculated from each wavelength, commonly known as autoscaling or standardization [34]. More formally this is calculated as
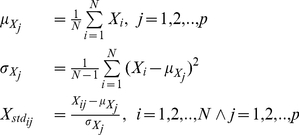
(1)



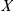
 is here a matrix of size 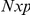 containing all the spectra in the calibration, where 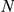 is the number of samples and 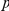 is the number of sampled wavelengths. A pixel-wise astaxanthin prediction of the images was done. The loadings of the astaxanthin PLS model (as described above) were used to project acquired preprocessed images into a subspace highlighting the distribution of the astaxanthin contentbased on electromagnetic reflection properties in each fillet. Principal Component Analysis (PCA) was used for visualizing trends in the multivariate dataset, and identifying outliers. All extraction, image analysis routines, color transformations, pixel based predictions and calibration analyses were programmed in Matlab 7.8 (The Mathworks Inc., Natick, MA, USA).

## Results and Discussion

### Reflection characteristics of astaxanthin

**Figure 3:**
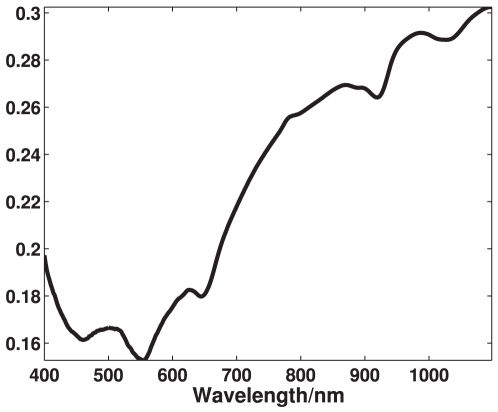
Reflection properties of astaxanthin. These reflection properties has been recorded using an absorbance spectrometer and transformed to reflection properties. The axes shows amount of light reflected as a function of wavelength.

The reflection spectrum recorded by the NIRSystems 6500 instrument, seen in Figure 3, shows large reflection properties starting from around 600 nm as well as large absorption properties from around 400 nm to 600 nm. This corresponds to having high absorption in the cyan, green and yellow area while the red and blue area is highly reflected, giving astaxanthin its characteristic dark red/purple color. The present measurements are well in accordance with previous absorption measurements of astaxanthin [35].

### Reference data

**Figure 4:**
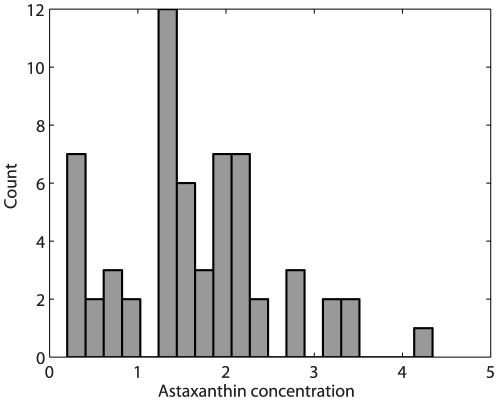
Distribution of measured astaxanthin. A histogram of the reference data shows the sample count as a function of 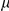
g astaxanthin per fish, revealing a high number of observations around 2
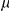
g.

The results from the chemical measurements of the astaxanthin content are presented in Figure 4. The astaxanthin concentration in the samples ranges from 0.2 to 4.34 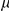
g per g fish with a mean of 1.69 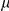
g per g fish and a standard deviation of 0.95 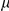
g per g fish.

### Acquired images and segmentation results

**Figure 5:**
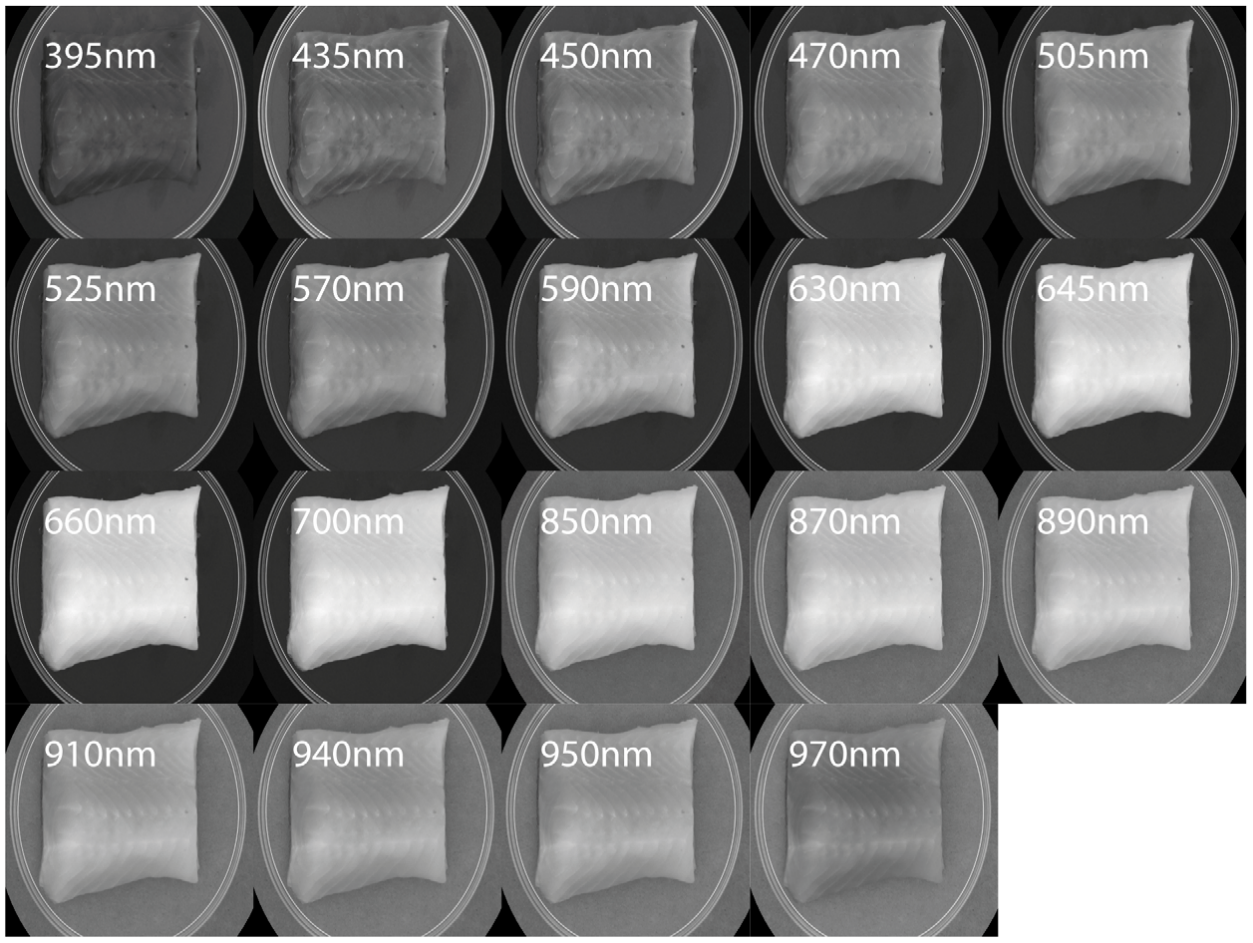
Channels ranging from 395 nanometer to 970 nanometer. A multispectral image of a trout fillet is here shown where the reflected light is seen for each narrowband LED, which gives a 19 dimensional spectrum for each pixel in the image.

**Figure 6:**
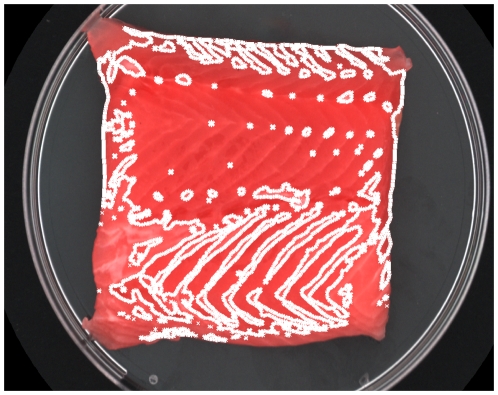
Example of fillet used in analysis with region of interest indicated as contours. The colors of the image are reconstructed from the multispectal image, while the mask is created using the maximum autocorrelation decomposition and otsu's threshold method.

An example of a recorded multispectral image is presented in Figure 5 with the channels listed according to their wavelength number in Table 1. It is clearly shown that the general brightness of the image increases as the wavelength increases and that some features are more pronounced at certain wavelengths that others e.g. fat and collagen in meat structure are primarily pronounced at low wavelengths (395 nm to 570 nm). An example of the final segmentation results is shown in Figure 6, where the mask indicates the segmentation of fillet from background the using the MAF transformation and fat the CDA transformation. The MAF transformation is a contrast between the extreme bands, ultra blue (385 nm) and NIR (970 nm), and the middle color bands in the blue/green area of the visible spectrum (630–700 nm). The segmentation of meat based on CDA transformation relies primarily on the blue part of the spectrum (430–470 nm).

### Extracted spectra

**Figure 7:**
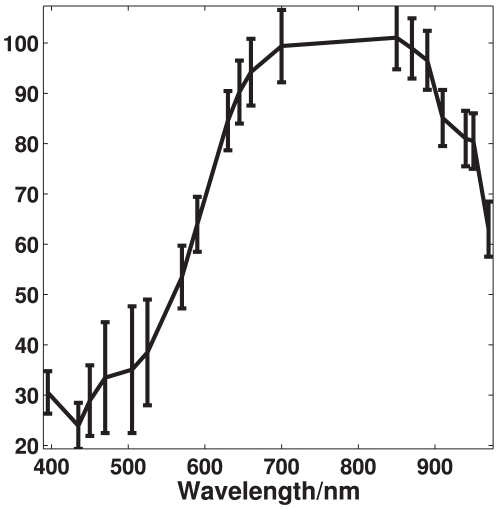
Autoscaling of mean spectra. The mean of all mean spectra is seen together with errorbars indicating one standard deviation of all mean spectra. Each mean spectrum is calculated as the mean of all pixels within the region of interest in a multispectral image.

**Figure 8:**
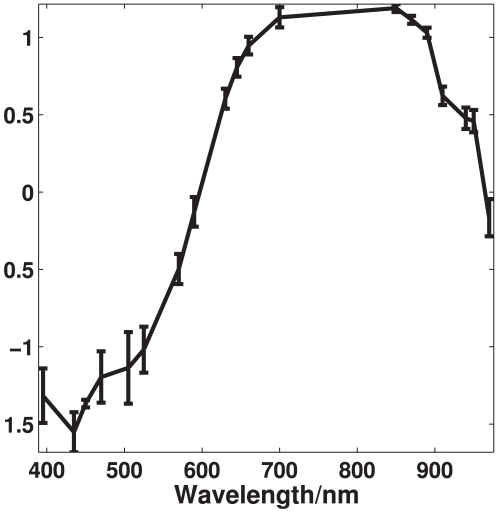
Autoscaling of mean spectra. Autoscaled spectra show significant lower variation in general except for the area between 400 and 500 nm which is now highlighted

Figure 7 shows the mean spectrum of each of the 59 recorded trout fillets. A general scaling difference is seen in the spectra which have been removed using autoscaling preprocessing, thereby highlighting the nonlinear differences. The scaling of a spectrum in the visible range is in general an expression for the brightness of the sample, which means some fish among the samples set appear brighter than other. A clear difference in the intensity of the spectra, which becomes very apparent after treating with autoscaling in Figure 8, is seen in the area around 450 to 525 nm. This area corresponds quite well to a known absorption area of astaxanthin, which also is seen in Figure 3. Further a deviation in the spectra is seen in the NIR area and below 435 nm.

### Calibration models

**Figure 9:**
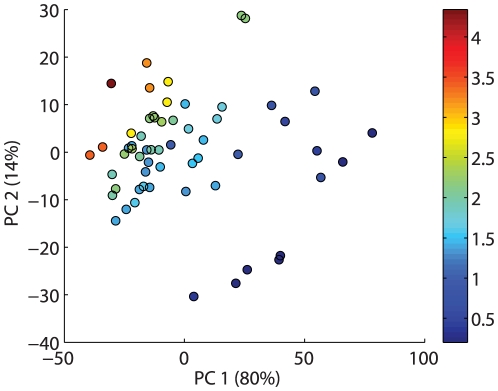
Directions of maximum variation in the autoscaled meanspectra. A scoreplot for a principal component analysis of the 19 dimensional mean spectra shows a definite trend in the the data along the first principal component which accounts for 80% of the variation in the dataset. The second principal component on the y axis accounts for a total of 14%. Each label in the plot represents a multispectral image of a trout fillet, and the number is the concentration in the corresponding fillet in 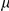
g.

**Figure 10:**
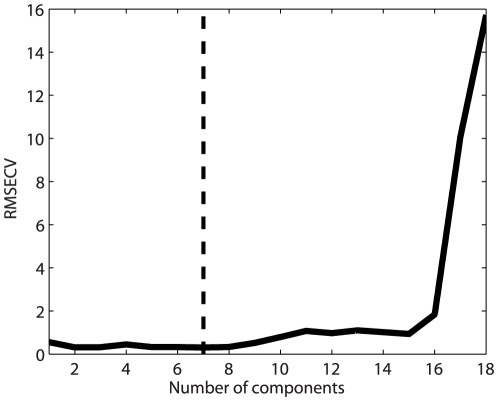
Generalization error calculated as RMSECV. To select a proper model, a leave one out cross validation scheme has been used, where the sum of squared errors (RMSECV) are shown here as a function of components included in the model. The lowest error is indicated with a vertical line, corresponding to a total of 7 components.

**Figure 11:**
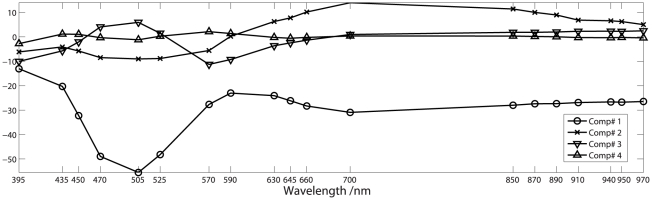
Loadings from PLS model of the multispectral images. The PLS model used to make astaxanthin predictions is based on seven loadings where the first four are shown here. Each loading is shown with a unique symbol, and indicates that especially the area in the beginning of the visible spectrum is important for astaxanthin prediction.

Figure 9 shows a score plot (PC1 versus PC2) of a PCA of the entire dataset after autoscaling. The plot shows a clear trend in the first component describing 80% of the variation with few outliers. Based on outlier diagnostics seven samples were categorized as outliers and removed from the data set prior to further analysis. All outliers were characterized by bad filleting. Table 2 shows the results of the final PLSR model for astaxanthin prediction based on a multispectral image. The reported RMSEP of 0.26 from a 7 component PLSR model is based on an independent test set while the model itself was cross validated on a trainingset. The cross validation showed a minimum generalization error when using 7 PLS components (Figure 10), which together with a total variance description on the response variable of 91% led to the choice of 7 components in the model. The variance decription percentages for the 7 components in the response variable were 48, 69, 75, 83, 86, 89 and 91% which shows that the performance of the model drastically increases with the first 4 components. The loadings for these components are shown in Figure 11. The first 2 components clearly show high response in the area around the absorption peak of astaxanthin in the blue/green area of the visual spectrum. The components naturally reflect the areas of largest variation in the preprocessed spectra shown in Figure 8. Table 2 also contain a PLSR model fitted on the same data after transformation to sRGB images. This means only a total of 3 variables exists for the regression problem which could basically be handled using a full multiple linear regression. This was tested together with a PLS model, which was found to yield equal results. The RGB model in Table 2 is seen to have a higher RMSEP value, indicating reduced prediction abilities than the multispectral mode. Among the two models the multispectral models has best variance description with an 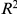 value of 0.86 versus and 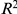 value of 0.66 for the RGB model. Furthermore the variance in the residuals is seen to be smaller for the multispectral model with a standard error of 0.02 against 0.05 for the RGB model. Previously similar techniques has been investigated using VIS spectroscopy and digital photography in [36], for calibration against chemically measured astaxathin. VIS spectroscopy was here found to have a correlation coefficient of 0.92 with a RMSEP of 0.42. For digital photography a correlation of 0.92 was found together with an RMSEP of 0.41. They reported a cross validation error based on all samples, meaning this error was used to chose the correct number of components. Compared to our results, we managed to achieve an RMSEP of 0.27 on an independent testset, to get a truly unbiased model, for the multispectral images.

**Table 2:** Prediction results obtained using multispectral and sRGB images.

	Multispectral	sRGb
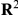 **(testset)**	0.86	0.66
**RMSEP (testset)**	0.27	0.45
**Std. Error (testset)**	0.02	0.05

**Figure 12:**
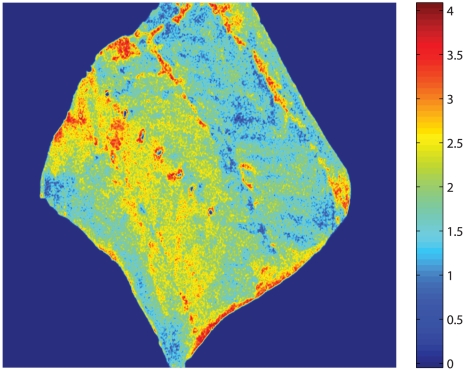
Projected PLS image. Chemically measured average astaxanthin content for the entire piece: 2.26
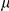
g The multispectral image is unfolded and projected using the loadings from calibrated prediction model. The result is reshaped to an image which then gives a spatial overview of the astaxanthin within the fillet, based on the light properties of the surface.

The calibrated PLS model makes it possible to predict the astaxanthin concentration in each pixel of the image - a so called pixel-wise prediction. The pixel-wise prediction can therefore be used to estimate the astaxanthin distribution in the fillet. An illustration of this is seen in Figure 12, where a multispectral image of a trout piece is projected pixel-wise, in order to get an impression of the spatial distribution of the astaxanthin concentration. The pixel-wise prediction is color-coded according to the amount of astaxanthin predicted in each pixel, so that pixels with high values of astaxanthin appears red, while low value astaxanthin pixels appear blue. The projected image clearly shows that the upper part of the fillet contain the largest concentration of astaxanthin. This technique is well suited for visualization purposes. However, since the PLS model in this study was based on the preprocessed mean spectra from the entire salmonid pieces, the accuracy of this pixel-wise predictions remains to be validated properly in a further study.

### Conclusion

In this paper an experiment was conducted to examine the possibilities of using multispectral imaging to assess the concentration of cartenoids, with focus on astaxanthin, in rainbow trout fillets. The recorded images ranged spectrally throughout the visible area and up into the first part of the near infra red area. The astaxanthin concentration of the investigated fillets ranged from 0.2 to 4.34 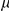
g per g fish with a mean of 1.69 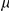
g per g fish. A total of 7 images were classified as outliers using PCA scoreplots for identification. A PLSR model was calibrated based on mean values of spectral value in a region of interest in the image. A training set was used for model training in a leave one out cross validation scheme, while a separate test set was used to evaluate the model in terms of RMSEP and and 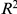. The result was compared to a similar model based on color images extracted from the multispectral images, in order to motivate the use of multispectral images in a study like this. As a consequence of offering more spectral information about the sample, it is possible to gain more knowledge about which area of the spectrum yields the information we are interested in. The RMSEP obtained from the test set was 0.27 for the multispectral images and 0.45 for the color images, showing a somewhat higher prediction certainty for the multispectral images. Furthermore, the goodness of fit (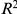) was similarly somewhat better for the multispectral model, being 0.86. The most significant components of the PLSR model revealed that the area between 470 and 525 nm. carried the largest amount of variation, which corresponds very well with absorption peaks of pure astaxanthin being in the vicinity of 450 to 600 nm. In conclusion, the current study has shown that multispectral imaging is a promising method for rapid analysis of the astaxanthin concentration of rainbow trout, and thereby a qualified candidate for replacement of ordinary laborious and destructive sampling of astaxanthin for concentration prediction.
